# First report on a novel *Nigrospora sphaerica* isolated from *Catharanthus roseus* plant with anticarcinogenic properties

**DOI:** 10.1111/1751-7915.12603

**Published:** 2017-06-14

**Authors:** Farah Wahida Ayob, Khanom Simarani, Nurhayati Zainal Abidin, Jamaludin Mohamad

**Affiliations:** ^1^ Institute of Biological SciencesFaculty of ScienceUniversity of Malaya50603Kuala LumpurMalaysia

## Abstract

This paper reports on the vinca alkaloid produced by a novel *Nigrospora sphaerica* isolated from *Catharanthus roseus*. Through liquid chromatography–mass spectrometry (LCMS), only the crude mycelia extract of this fungus was positive for determination of vinblastine. This vinca alkaloid was then purified by using high‐performance liquid chromatography (HPLC) and tested for cytotoxicity activity using MTT assays. The breast cell line cancer (MDA‐MB 231) was treated with a purified vinblastine which was intracellulary produced by *N. sphaerica*. The purified vinblastine from extracted leaf of *C. roseus* was used as a standard comparison. A positive result with a value of half maximal inhibitory concentration (IC_50_) of > 32 μg ml^−1^ was observed compared with standard (IC_50_) of 350 μg ml^−1^ only. It showed that a vinblastine produced by *N. sphaerica* has a high cytotoxicity activity even though the concentration of vinblastine produced by this endophytic fungus was only 0.868 μg ml^−1^.

## Introduction

Endophytes are microbes that refer to microorganisms that live inside the tissues of plants without causing any apparent harm or diseases to the host plant (Strobel, 2002). In fact, they promote the host plant's growth and the formation of secondary metabolites related to the plant defence (Petrini, 1991 & Chandra *et al*., 2010). They could produce valuable bioactive compounds with a varied application in both of research and applied fields (Ravindra *et al*., 2014). Endophytic fungi spend the whole part of their life cycle living symbiotically within the healthy tissues of the host plant (Tan and Zhou, 2001; Ravindra *et al*., 2014). It also has been recognized as one of important and novel resources of natural bioactive products (Strobel *et al*., 2004) as most endophytes are capable of synthesizing bioactive compounds that may provide plants with a defence against pathogens (Guo *et al*., 2008). Some of these compounds have proven useful for discovering a novel drug (Yan *et al*., 2011). There are many reports that endophytic fungi isolated from a medicinal plant produce a new drug or compound that similar to the host plants (Table 1). All these findings will help to fill the demands of the drugs. In fact, the manufacturing cost of the drugs from endophytic fungi is cheaper than production from the plants as it takes a shorter period to produce it.

**Table 1:** The list of previous research on endophytic fungi which produced a drug

Host plants	Endophytic fungi	Drugs	References
*P. amarus*	*Trichothecium* sp.	Trichothecinol‐A	(Ravindra *et al*., 2014)
*Eugenia jambolana*	*Cephalotheca faveolata*	Sclerotiorin	(Periyasamy *et al*., 2012)
*Camptotheca acuminate*	*Fusarium solani*	Camptothecin	(Lin *et al*., 2011;
*Taxus brevifolia*	*Taxomyces andreanale*	Taxol	(Wani *et al*., 1971)
*Podophyllum peltatum*	*Phialocaphala fortinii*	Podophyllotoxin	(Kumar *et al*., 2013)

*Catharanthus roseus* or well known as a Madagascar periwinkle is a medicinal plant belonging to the family Apocynaceae (Gajalakshmi *et al*., 2013). Even though this plant is native to Madagascar, it also can be found in Malaysia. Here, it is called as Kemunting Cina and the flower of this plant was chosen as a logo for the National Cancer Council Malaysia (MAKNA) (Ayob and Simarani, 2016). This plant also well known to produce a lot of important compounds especially vinca alkaloids vinblastine and vincristine (Manganey *et al*., 1979; Krishnan, 1995). Besides, this plant also produces vindoline and catharanthine which are the major monomer alkaloids as well as a biosynthetic precursor for vinblastine and vincristine (Noble, 1990). In 1960, vinblastine was introduced to treat certain types of cancer including breast cancer, testicular cancer and Hodgkin's disease (Armstrong *et al*., 1964), while in 1963, vincristine was introduced through oxidization of vinblastine to treat leukaemia (Evans *et al*., 1963). So far, there are only three reports on these alkaloids produced by the endophytic fungi, which were *Alternaria* sp., *Fusarium oxysporum* and unidentified fungi from *C. roseus* (Guo *et al*., 1998; Zhang *et al*., 2000; Yang *et al*., 2004). Thus, this research was carried out to find a novel endophytic fungus that could produce vinca alkaloids, vinblastine and vincristine from the host plant *C. roseus*. The purified alkaloids will be tested for cytotoxicity test through MTT assay against breast cell line cancer.

## Results and discussion

**Figure 1:**
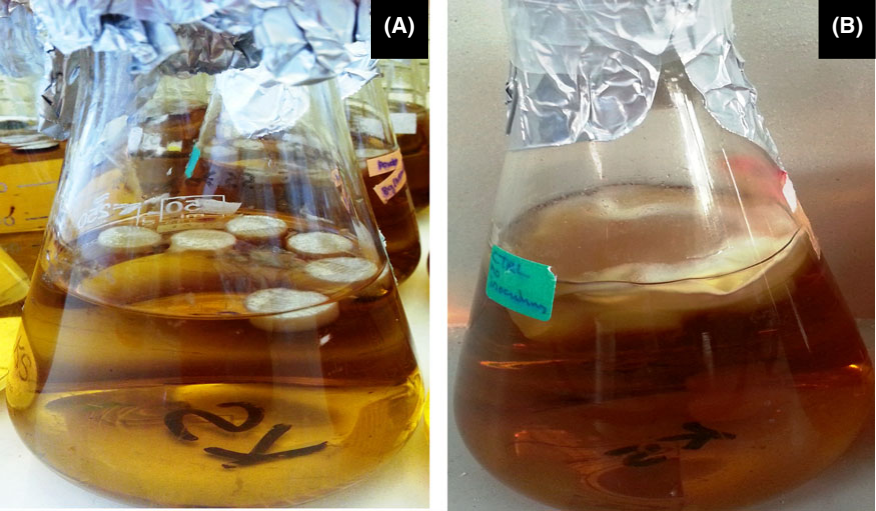
The crude fungal extract of *N. sphaerica* was prepared using broth culture technique where the inoculum was cultured into a yeast extract sucrose broth (YESB) medium and incubated at 25 °C under static condition (A). After 21 days, the cultured can be harvested using filter separation method (B).

**Figure 2:**
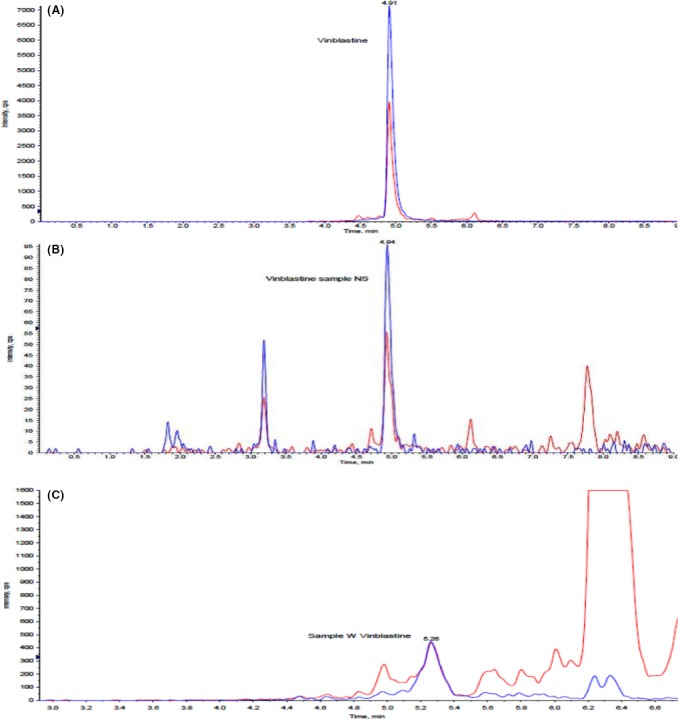
The liquid chromatography–mass spectrometry (LCMS) chromatograph of commercial standard vinblastine (A), crude mycelia extract of *N.sphaerica* isolated from *C. roseus* plant (B) and crude leaf extract of *C. roseus* (C) at retention time of 4.91, 4.94 and 5.2 min, respectively. Symbol: Blue line = First fragment ion, Red line = Second fragment ion.

**Figure 3:**
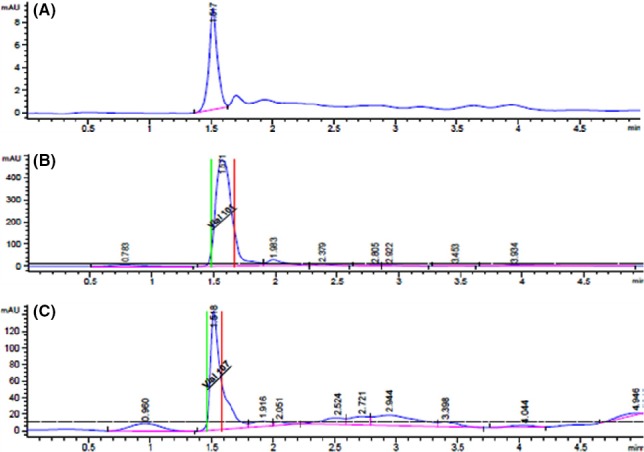
The high‐performance liquid chromatography (HPLC) chromatograph of commercial standard vinblastine (A),vinblastine produced by the crude mycelia extract of *N. sphaerica* isolated from *C. roseus* plant (B) and crude leaf extract of *C. roseus* (C) has been purified at retention time of 1.5–1.6 min.

The crude fungal extract was prepared using the 21 days of fungal cultured (Fig. 1), while the crude leaf extract was prepared using a dried leaves of wildly grown *C. roseus* (white). Through a liquid chromatography–mass spectrometry (LCMS), the crude leaf extract showed a presence of both vinblastine and vincristine. However, crude mycelia extract only showed a positive result on vinblastine. It was determined by the presence of two fragment ions from a peak in the same compounds and indicated a confirmation of the compound detected (Fig. 2). Meanwhile, no detected peak was observed for both alkaloids in a crude broth fungal extract. This result indicated that the vinblastine was intracellularly produced by this fungus. The crude mycelia extract of *N. sphaerica* fermented in yeast extract sucrose broth (YESB) and crude leaf extract of *C.roseus* consisted 0.868 and 0.666 μg ml^−1^ of vinblastine respectively (Fig. 3). Hence, in this study, the cytotoxicity test was only carried out using vinblastine due to the absence of vincristine in the extracted fungal mass sample.

**Figure 4:**
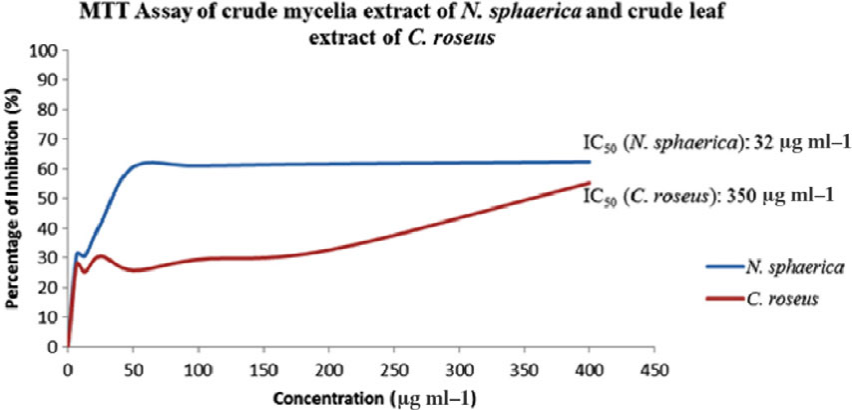
The MTT assay of vinblastine produced by the crude mycelia extract of *N.sphaerica* isolated from *C. roseus* plant and crude leaf extract of *C. roseus* against breast cell line cancer MDA‐DB 231 with various concentration 6.35–400 μg ml^−1^.

**Figure 5:**
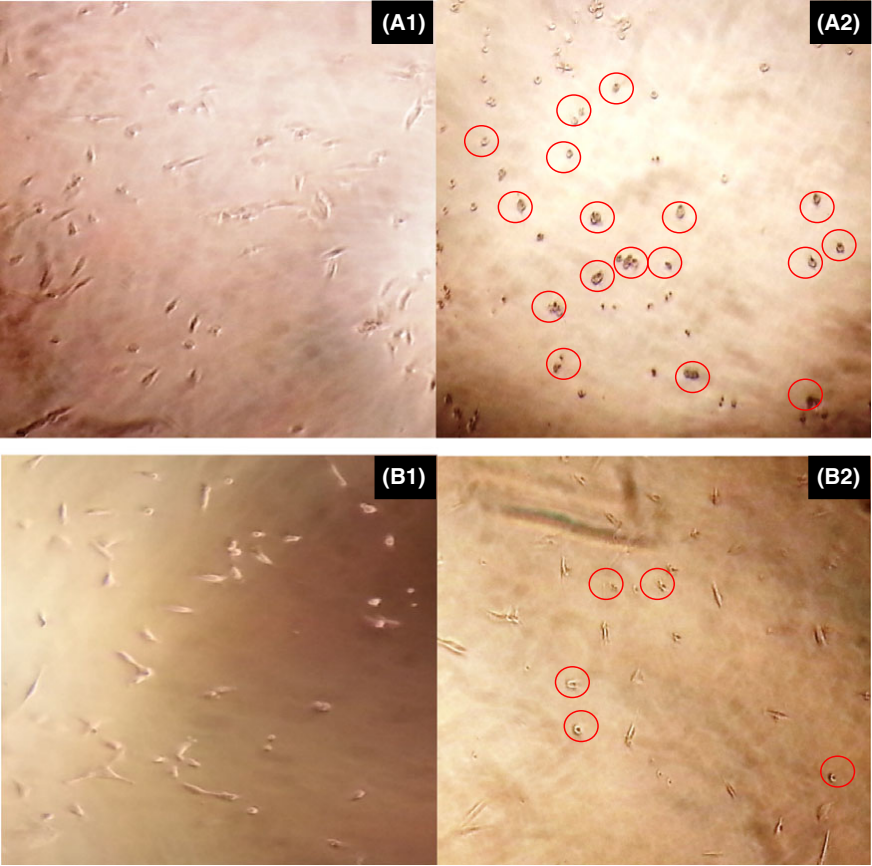
The structure of breast cell line cancer MDA‐DB 231 before treated with a crude mycelia extract of *N. sphaerica* (A1) and a crude leaf extract of *C. roseus* (B1). After 72 h treated with different concentrations of vinblastine produced by both crude extracts, the structure of breast cell line changed from a rod shape into round shape (red circle). The breast cell line cancer treated with a crude mycelia extract of *N. sphaerica* gave a highest percentage of inhibition where most of the cells were died (A2) compared with the cell which treated with a crude leaf extract of *C. roseus* (B2).

Purified vinblastine from crude mycelia extract of this fungus and crude leaf extract were obtained using high‐performance liquid chromatography (HPLC). The samples were eluted for 5 min of retention time when loaded on analytical C_18_ column with a flow rate of 1 ml min^−1^. It was clearly seen from the chromatograph of HPLC, the retention time for fungal vinblastine peak was similar with vinca alkaloid extracted from leaf of *C. roseus*. Cytotoxicity of purified vinblastine produced by both endophytic fungus *N. sphaerica* and *C. roseus* was tested against human breast carcinoma MDA‐MB 231 cell line cancer with various concentrations (6.35–400 μg ml^−1^). The test showed a positive result with a half maximal inhibitory concentration (IC_50_) value of > 32 and 350 μg ml^−1^ for the vinblastine that has been purified from both crude fungal extract and a crude leaf extract respectively (Figs 4 and 5). This happened when the vinca alkaloid was binding to beta‐tubulin and disruption of microtubule function during mitosis, which leads to mitosis arrest and cell death (Damen *et al*., 2010). Jordan and Wilson (2004) in their reports also mentioned that the anticancer activity of these alkaloids was attributed to their ability to disrupt microtubules metaphase arrest in dividing cell.

Currently, the commercial vinblastine in the market is produced by *C. roseus*. However, it takes almost one year before it is ready for harvesting. In the other hand, vinblastine produced by *N. sphaerica* only takes only a month of preparation (cultivation, extraction and purification) before it is ready to be used. In short, the vinblastine could be produced faster with a huge amount using crude mycelia extract of *N. sphaerica* instead of current resource of this alkaloid, *C. roseus*. This new finding will help to fill the demand of this valuable natural product for cancer treatment. This was supported by Ravindra *et al*. (2011) that among the metabolites produced by the endophytic fungi, attention has attracted to the compound with anticancer properties. So far, there were 100 anticancer substances classified into 19 different chemical classes with an activity against 45 different cancer cell lines that have been isolated from 50 fungal species (Abdulmyanova *et al*., 2015). However, it was reported that only one fungal produced vinblastine; *Alternia* sp. which was isolated from the *C. roseus*. (Guo *et al*., 1998).

As a conclusion, the crude mycelia extract of *N. sphaerica* isolated from a medicinal plant *C. roseus* was positive produced vinblastine with a concentration of 0.868 μg ml^−1^ which is higher than vinblastine produced by the crude leaf extract (0.666 μg ml^−1^). The cytotoxicity test using the 3‐(4, 5‐dimethylthiazol‐2‐yl)‐2, 5‐diphenyltetrazolium bromide (MTT) assay against breast cell line cancer MDA‐MB 231 for the vinblastine produced by both extracts showed that the vinblastine that has been purified from a crude mycelia extract of *N. sphaerica* has a better half maximal inhibitory concentration (IC_50_) > 32 μg ml^−1^ compared with the vinblastine purified from a crude leaf extract of *C. roseus* > 350 μg ml^−1^.

## Experimental procedures

### Microorganism and growth condition

The fungal strain used in this study was previously isolated from a *C. roseus* plant (white), purified and identified as *Nigrospora sphaerica* (Ayob and Simarani, 2016). The strain was maintained on slanting agar and reactivated on the PDA plate prior to use. There were six pieces of the plug (1 cm) of fungal growth culture, which were inoculated into a 250 ml conical flask containing 250 ml of yeast extract sucrose broth (YESB) medium (20 g of yeast extract, 40 g of sucrose and 1 l of distilled water with a pH 5.8). The flasks were then incubated at 25 °C under static condition for 21 days. The grown culture was harvested by filter separation method and used for crude extract preparation.

### Preparation of crude fungal extract and crude leaf extract

The crude fungal extract (CFE) from cell free filtrate and mycelia biomass were prepared according to Wiyakrutta *et al*. (2004) with minor modification. The cell free filtrate was extracted thrice with a dichloromethane (200 ml) and evaporated to dryness using rotary evaporator. The extracted sample was weighed to constitute the crude filtrate extract (CFE). Meanwhile, the mycelia biomass were freeze‐dried and extracted twice with a mixture of dichloromethane:methanol (1:1, v/v) for 1 h. The extracted sample was then air‐dried and weighed before kept in the airtight container until further used.

Meanwhile, the crude extracts of *C. roseus* leaves were prepared according to Gupta *et al*. (2005). Briefly, 5 g of powdered samples was soaked thrice with 90% (v/v) ethanol (3 × 30 ml) for 12 h each at room temperature. The alcohol extract was filtered and concentrated in vacuo to reduce the volume down to 10 ml. The sample was then diluted with 10 ml of distilled water followed by acidified with 10 ml of 3% (v/v) hydrochloric acid and washed with hexane (3 × 30 ml). The aqueous portion was basified with ammonia to pH 8.5 and extracted using chloroform (3 × 30 ml). The chloroform extract was washed with distilled water, dried over sodium sulfate and concentrated under vacuum before dissolving in 10 ml of methanol. Both of crude extracts were then dissolved separately in dimethylsulfoxide (DMSO) to obtain 1 g ml^−1^ of concentration.

### Analysis/determination of vinca alkaloids

The presence of vinca alkaloids in both crude extracts were detected using liquid chromatography–mass spectrometry (AB Sciex 3200Q Trap LCMS/MS; AB Sciex Pte,Ltd., Ontario, Canada), method multiple reaction monitoring (MRM), equipped with a column Agilent Zorbax XDB C18 (150 × 4 × 5 μM) and buffered with (A) pure water, ammonium formate and formic acid, (B) acetonitrile, ammonium formate and formic acid. The experiment was run for 10 min and rapidly screened for the peaks because it should have two fragment ions from the same compounds for further confirmation of the peaks.

The fungal crude extracts were then purified using high‐performance liquid chromatography (Agilent 1220 Infinity Gradient LC – G4294B) for vinblastine and vincristine. The 10 μl sample was injected in HPLC column C18 Merck (50 × 2 × 1.5 μM), and isocratic elution was performed using methanol and Nano pure water with 0.01% acetic acid at a flow rate of 1 ml min^−1^. dual wavelengths 254 and 260 nm were used to detect the compound eluting from the column. The purified vinblastine was collected using Agilent Fraction Collector (G1364C) and was tested for cytotoxicity test.

### Cytotoxicity activity (MTT assay)

Cytotoxicity of vinblastine was assessed using the 3‐(4, 5‐dimethylthiazol‐2‐yl)‐2, 5‐diphenyltetrazolium bromide (MTT; Sigma) assay against breast cell line cancer MDA‐MB 231. The cancer cell were grown in Dulbecco's modified Eagle's medium (DMEM) and washed in phosphate‐buffered solution (PBS) before 100 000 cells were placed in each well of 96‐well plates and treated with fungal extract. After 72 h incubated at 37 °C and 5% CO_2_, the MTT reagent was removed from the plate and replaced with a DMSO and gently shaken for 30 min. The absorption was determined at 570 nm. The percentage of inhibition (POI) was calculated using formula:

(1) $$\begin{array}{cc} \text{POI}= & \frac{\left(\text{Absorbance of control}-\text{Absorbance of sample}\right)}{\text{Absorbance of control}}\\ & \times 100 \end{array}$$

The half maximal inhibitory concentration (IC_50_) was determined from the graph of samples concentration vs. percentage of inhibition.

## Conflict of interest

None declared.
